# CircPTPN22 modulates T-cell activation by sponging miR-4689 to regulate S1PR1 expression in patients with systemic lupus erythematosus

**DOI:** 10.1186/s13075-023-03150-3

**Published:** 2023-10-19

**Authors:** Zhuyan Jiang, Shifei Li, Yuying Jia, Qijun Wu, Xuemeng Chen, Mengjie Zhang, Qingqing Miao, Zhiting Zhong, Zhifang Zhai, Bing Ni, Jun Xiao, Jun Tang

**Affiliations:** 1 Dermatology Department of The First Affiliated Hospital of USTC, Division of Life Sciences and Medicine, University of Science and Technology of China, Hefei, Anhui 230001 China; 2 Department of Dermatology, Southwest Hospital, Third Military Medical University, Chongqing, 400038 China; 3 PLA Clinical College, Anhui Medical University, Hefei, Anhui 230001 China; 4 Department of Rheumatology, Southwest Hospital, Third Military Medical University, Chongqing, 400038 China; 5 Department of Pathophysiology, Third Military Medical University, Chongqing, 400038 China; 6 Intelligent Pathology Institute, Division of Life Sciences and Medicine, University of Science and Technology of China, Hefei, 230036 Anhui China; 7 Department of Cardiovascular Medicine, Chongqing University Central Hospital, Chongqing, 400014 China

**Keywords:** Circular RNAs, MicroRNAs, CeRNA, Systemic lupus erythematosus, T cells

## Abstract

**Background:**

Circular RNAs are involved in autoimmune disease pathogenesis. Our previous study indicated that circPTPN22 is involved in autoimmune diseases, such as systemic lupus erythematosus (SLE) and rheumatoid arthritis, but the underlying mechanisms remain unclear.

**Methods:**

First, the expression of circPTPN22 was detected by real-time PCR and western blotting. After overexpression or knockdown of circPTPN22, the proliferation of Jurkat cells was detected by the CCK-8 assay, and the apoptosis of Jurkat cells was detected by flow cytometry. In addition, the relationship between circPTPN22-miR-4689-S1PR1 was confirmed by bioinformatic analyses, fluorescence in situ hybridization assays, RNA-binding protein immunoprecipitation, and dual luciferase reporter assays.

**Results:**

We found that circPTPN22 expression was downregulated in the PBMCs of SLE patients compared to those of healthy controls. Overexpression of circPTPN22 increased proliferation and inhibited apoptosis of Jurkat T cells, whereas knockdown of circPTPN22 exerted the opposite effects. CircPTPN22 acts as a miR-4689 sponge, and *S1PR1* is a direct target of miR-4689. Importantly, the circPTPN22/miR-4689/S1PR1 axis inhibited the secretion of TNF-α and IL-6 in Jurkat T cells.

**Conclusions:**

CircPTPN22 acts as a miR-4689 sponge to regulate T-cell activation by targeting *S1PR1*, providing a novel mechanism for the pathogenesis of SLE.

**Supplementary Information:**

The online version contains supplementary material available at 10.1186/s13075-023-03150-3.

## Introduction

Systemic lupus erythematosus (SLE) is a chronic autoimmune disease characterized by the loss of autotolerance and production of antibodies against autoantigens [1]. The clinical manifestations of SLE are diverse and involve several organs and tissues, such as the skin, kidneys, joints, and nervous system [2]. SLE pathogenesis is associated with genetic, environmental, hormonal, epigenetic, and immunomodulatory factors [3]. Altered T-cell function has been reported to be critical in the pathophysiology of SLE as these cells react with other immune cells to activate and amplify inflammatory processes, leading to tissue damage [4, 5]. Therefore, it is essential to further explore the mechanisms of T cells in SLE to achieve better outcomes.

Circular RNAs (circRNAs) are a class of covalently closed noncoding RNAs (ncRNAs) that play crucial roles in transcriptional and posttranscriptional regulation in eukaryotic cells [6]. Recently, circRNAs were found to function as competing endogenous RNAs (ceRNAs) of microRNAs (miRNAs) and inhibit their function [7]. MiRNAs are a class of noncoding RNAs that are approximately 22 nt long and can be translated by interacting with the mRNA 3'-untranslated region (3'-UTR) to form an RNA-induced silencing complex (RISC). RISC can inhibit mRNA translation or degradation to achieve posttranscriptional regulation [8]. The parent gene of circPTPN22, protein tyrosine phosphatase nonreceptor 22 (PTPN22), involved in several autoimmune diseases, is considered a candidate susceptibility gene for SLE, and has potent regulatory functions in T-cell activation [9–11]. In our previous study, circPTPN22 was identified as a potential new activity indicator in peripheral blood mononuclear cells (PBMCs) in SLE [12]. However, elucidating the specific mechanism of circPTPN22 in T cells of SLE requires further investigation.

In this study, we explored whether circPTPN22 is involved in SLE pathogenesis by sponging its target miRNAs to affect T-cell functions in vitro. We found that circPTPN22 sponges miR-4689, affects the proliferation and apoptosis of Jurkat T cells and decreases the secretion of tumor necrosis factor-alpha (TNF-α) and interleukin-6 (IL-6) via a ceRNA mechanism in Jurkat T cells. Moreover, 1-sphingosine receptor 1 (*S1PR1)* is a direct target of miR-4689. Therefore, our results demonstrated the modulatory functions of circPTPN22 in T-cell activation by sponging miR-4689 to target *S1PR1* in SLE pathogenesis.

## Materials and methods

### Patients and clinical samples

A total of 45 patients with SLE and 44 age- and sex-matched healthy controls (HCs) were recruited from Southwest Hospital, the First Affiliated Hospital of Third Military Medical University (Chongqing, China), between October 2019 and September 2020. The healthy donors had no history of autoimmune diseases or immunosuppressive therapy. Patients with concurrent infections were excluded from the study. All the participants fulfilled the American College of Rheumatology (ACR) classification criteria for SLE. Detailed information on the characteristics of all participants is presented in Table 1. The study was approved by the Research Ethics Committee of Southwest Hospital, First Affiliated Hospital of the Third Military Medical University (approval no. KY2019119). Informed consent was obtained from all the participants.

**Table 1:** Main clinical and laboratory parameters of objections

Parameters	SLE	Healthy control
*n*	44	45
Age	30.5 ± 1.6	31.8 ± 1.5
SLEDAI	9.3 ± 3.8	NA
Disease duration (month)	17.9 + 1.9	NA
Skin involvement, P/N (*n*)	30/15	NA
Alopecia, P/N (*n*)	12/33	NA
Arthritis, P/N (*n*)	24/21	NA
Lupus nephritis, P/N (*n*)	20/25	NA
Leukopenia, P/N (*n*)	7/38	NA
Anti-ANA, P/N (*n*)	43/2	NA
Anti-dsDNA, P/N (*n*)	20/25	NA
Anti-Sm, P/N (*n*)	12/33	NA
C3 or C4 deficiency, P/N (*n*)	31/14	NA
Medication		
None	3	NA
Prednisone ≤ 30 mg/day	22	NA
Prednisone > 30 mg/day	20	NA

*P/N* positive/negative, *NA* not applicable

### Isolation of PBMCs

PBMCs were isolated from the SLE patients and healthy controls according to the human lymphocyte isolation solution protocol (TBD, Tianjin, China) by gradient centrifugation at 1800 × *g* for 30 min at room temperature. Cells in the interphase were collected and washed twice with Hank’s balanced salt solution (HBSS; HyClone, Logan, UT, USA). PBMCs in 1 mL TRIzol (Invitrogen, Waltham, MA, USA) and serum samples were stored at − 80 °C for subsequent experiments.

### Cell culture and transfection

Jurkat T cells were cultured in RPMI 1640 (Gibco, Waltham, MA, USA), and HEK-293 T cells were cultured in Dulbecco’s modified Eagle’s medium (DMEM; Gibco) supplemented with 10% fetal bovine serum (Gibco) and 1% penicillin/streptomycin (Gibco) and maintained at 37 °C and 5% CO_2_. The miRNA mimics, inhibitors, and controls were purchased from Ruibo Biosciences (Guangzhou, China). pcDNA3.1 lentivirus products LV-circRNA, sh-circRNA, and the corresponding negative controls were constructed in Jurkat T cells. Stably transfected cells were verified by quantitative real-time PCR (qRT‒PCR).

### Cell counting kit-8

Cell proliferation was measured using a standard cell counting kit-8 (CCK-8; Dojindo, Kumamoto, Japan) according to the manufacturer’s instructions. Briefly, 6 × 10^3^ cells were plated in 96-well plates and cultured for 0, 24, 48, and 72 h in an incubator. A total of 10 μL CCK-8 solution was added to each well and incubated at 37 °C for 2 h. Absorbance was measured at 450 nm using a standard microplate reader.

### Apoptosis assays

Cell apoptosis was determined using the Annexin V Apoptosis Detection Kit PE (Thermo Scientific, Waltham, USA) according to the manufacturer’s instructions. Briefly, 6 × 10^5^ cells were harvested and resuspended in 500 μL of 1 × binding buffer. After adding 5 μL Annexin V-PE and 10 μL 7-aminoactinomycin D (7-AAD) to each tube, the cells were incubated in the dark at room temperature for 10 min. Then, the samples were analyzed using flow cytometry (BD Biosciences, San Jose, CA, USA).

### Cell cycle analysis

Cell cycle progression was determined using a cell cycle detection kit (MULTI SCIENCES, Hangzhou, China). Briefly, 5 × 10^5^ cells were harvested, washed twice with phosphate-buffered saline (PBS), collected by centrifugation at 300 × *g* for 5 min at room temperature, and treated with 10 μL permeabilization solution and 1 mL DNA staining solution. Finally, the cells were incubated for 30 min in the dark at room temperature and analyzed using a flow cytometer (BD Biosciences, San Jose, CA, USA).

### CeRNA analysis and target prediction

We constructed a circPTPN22-miRNA‒target gene network using Cytoscape software (https://cytoscape.org) to visualize interactions based on high-throughput RNA sequencing data. In the network, the circRNA-miRNA interaction was analyzed using the databases of RNAhybrid (bibiserv.techfak.uni‑bielefeld.de/rnahybrid) and miRanda (http://www.microrna.org/microrna/home.do). MiRNA target genes were predicted using the TargetScan (http://www.targetscan.org/vert_71/), miRDIP (http://ophid.utoronto.ca/mirDIP/), miRDB (http://mirdb.org) and miRWalk (http://mirwalk.umm.uni-heidelberg.de) databases. A protein‒protein interaction (PPI) network was constructed using the STRING database (https://string-db.org/).

### RNA extraction and quantitative real-time PCR

Total RNA was extracted using TRIzol reagent (Invitrogen, Waltham, MA, USA) according to the manufacturer’s protocol. Subsequently, RNA concentration and purity were measured using a NanoDrop spectrophotometer (Invitrogen, Waltham, MA, USA) and 1% agarose gel. Total RNA (1000 ng) was reverse-transcribed into complementary DNA (cDNA) using a PrimeScript RT reagent kit with gDNA Eraser (Takara Bio, Beijing, China). Quantitative real-time PCR was performed using cDNA and SYBR Premix Ex Taq II (Takara Bio, Beijing, China) in a CFX96 Thermal Cycler (Bio-Rad, Shanghai, China) following the manufacturer’s instructions. The relative expression levels were calculated using the 2^−△△Ct^ method. The primers used are listed in Table 2, and the miRNA reverse primers used were universal primers from the Mir-X miRNA qRT‒PCR TB Green Kit (Takara Bio, Beijing, China).

**Table 2:** Primer sequences for RT‒PCR

Gene	Sequence (5'-3')
circPTPN22	F: AATTCTCACCAAATGTTCCCA
	R: AAGGTACATCATGGTCTGGC
S1PR1	F: GCCTCTTCCTGCTAATCAGCG
	R: GCAGTACAGAATGACGATGGAG
GAPDH	F: GGAGTCCACTGGCGTCTTC
	R: GCTGATGATCTTGAGGCTGTTG
miR-4689	F: TTGAGGAGACATGGTGGGGG
U6	F: CGCAAGGATGACACGCAAATTC

*F* forward, *R* reverse

### Enzyme-linked immunosorbent assay (ELISA)

Jurkat T cells were stimulated with IL-2 (50 ng/mL) for 30 min, and then human TNF-α and IL-6 in the Jurkat T-cell supernatant and serum in patients and healthy controls were measured by enzyme-linked immunosorbent assay (ELISA) according to the manufacturer’s protocol (Elabscience, Wuhan, China).

### RNA-binding protein immunoprecipitation (RIP) assay

The RIP assay was performed using an RNA Immunoprecipitation Kit (BersinBio, Guangzhou, China). The cell lysate was incubated with RIP buffer containing magnetic beads conjugated with Argonaute-2 (AGO2) or IgG control antibody (Abcam, Waltham, MA, USA). The expression of circPTPN22 in immunoprecipitates was determined by real-time PCR.

### Dual-luciferase activity assay

The wild-type or mutated binding sites of miR-4689 in the 3'-untranslated region (3'-UTR) of *S1PR1* mRNA and full-length circPTPN22 were cloned into a luciferase reporter vector (psiCHECK2). HEK-293 T cells were seeded in 96-well plates at a density of 5 × 10^3^ cells/well and incubated overnight. Then, the recombinant constructs were transfected into HEK-293 T cells together with miR-4689 or NC mimic using Lipofectamine 3000 (Invitrogen, Waltham, MA, USA). Firefly and Renilla luciferase activities were measured 48 h after transfection using a Dual-Luciferase Reporter Assay System (Promega, Madison, WI, USA) according to the manufacturer’s instructions using Promega GloMax 96 (Promega, Madison, WI, USA).

### Fluorescence in situ hybridization (FISH)

The expression of circPTPN22 and miR-4689 was detected using a FISH kit (RuiBoBio, Guangzhou, China) according to the manufacturer's instructions using a specific Cy3-labelled mix and Fam-labelled DNA oligo probes, respectively. Briefly, the cells were fixed with 4% paraformaldehyde for 15 min at room temperature and permeabilized with 0.25% Triton X-100 for 15 min at 4 °C. In situ hybridization was performed overnight at 37 °C using 20 μM circPTPN22 or miR-4689 probes in hybridization buffer. Then, the cells were washed continuously with 4 × , 2 × , and 1 × saline-sodium citrate buffer. Finally, nuclei were stained with DAPI, and images of the cells were captured using a Leica SP5 confocal microscope (Leica Microsystems, Mannheim, Germany).

### Western blot analysis

Cells were treated with cell lysis buffer containing 1 µL of protease inhibitor and 2 µL of phosphate inhibitor. After denaturation, 30 µg of protein in the loading buffer was subjected to SDS‒PAGE for isolation and then transferred to PVDF membranes. Membranes were blocked with 5% bovine serum albumin for 1 h at room temperature. Primary antibodies against S1PR1 (rabbit, 1:1000) and GAPDH (rabbit, 1:1000) from Thermo Fisher Scientific (Invitrogen, Waltham, MA, USA) were added and incubated with the PVDF membranes at 4 °C overnight and specific secondary antibodies for 1 h at room temperature. The protein band intensities were detected using an enhanced chemiluminescence kit (Thermo Scientific, Waltham, USA).

### Statistical analysis

One-way ANOVA or Student’s *t* test was used to analyze the data. SPSS 26.0 and GraphPad software (version 8.0) were used for the statistical analysis. Statistical significance was set at *P* < 0.05.

## Results

### Expression of circPTPN22 in patients with SLE and its potential clinical value

**Fig. 1:**
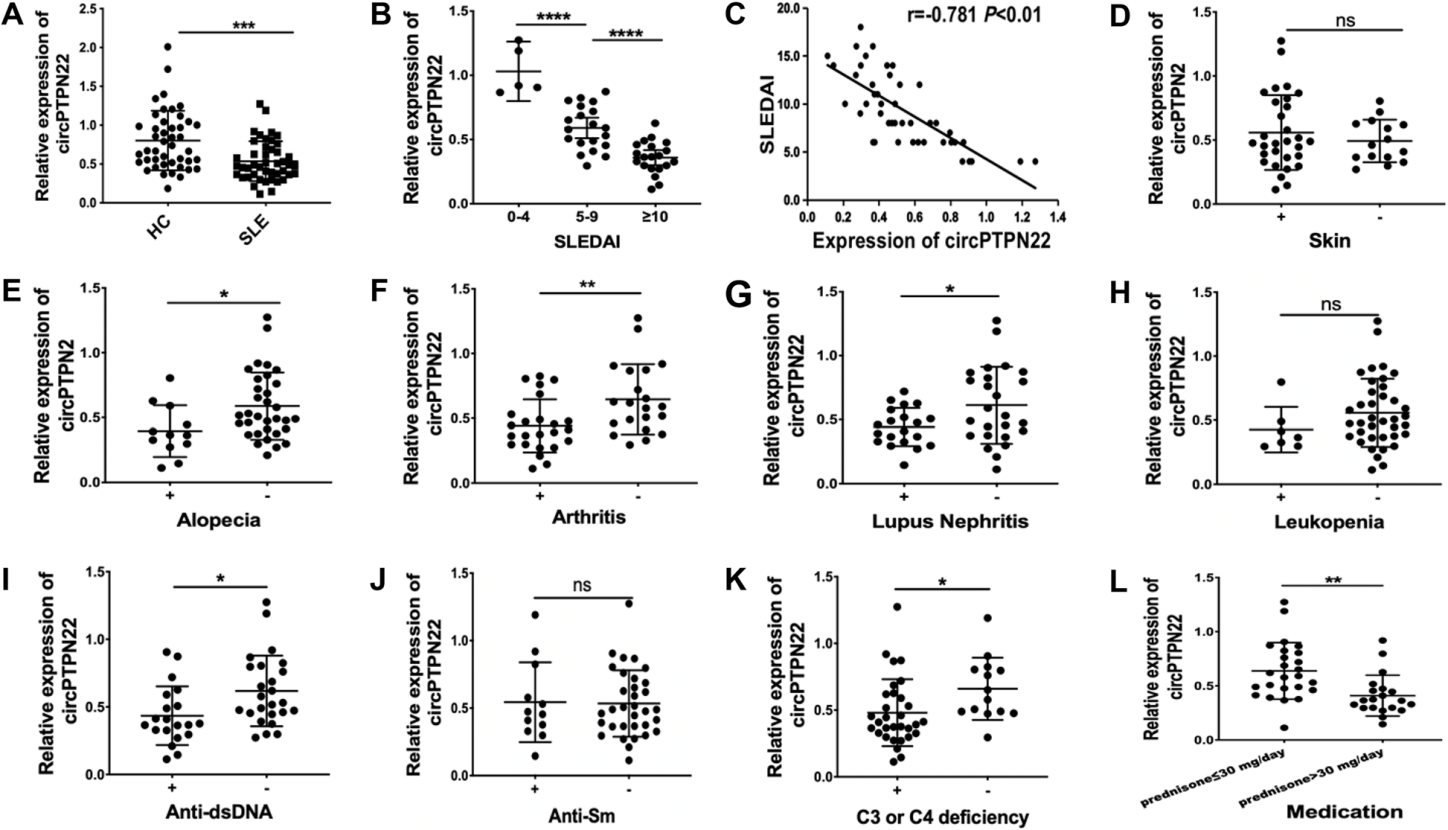
Expression of circPTPN22 in patients with SLE and its clinical relevance.** A** Relative expression of circPTPN22 in 44 HCs and 45 patients with SLE. **B** CircPTPN22 expression was lower in patients with SLE and higher SLEDAI scores. **C** Correlation analysis of circPTPN22 expression and SLEDAI. The expression of circPTPN22 in patients with SLE, with or without skin involvement (**D**), alopecia (**E**), arthritis (**F**), lupus nephritis (**G**), leukopenia (**H**), anti-dsDNA ( +) (**I**), anti-Sm ( +) (**J**), and C3 or C4 deficiency (**K**), was compared. **L**. Expression of circPTPN22 among SLE patients receiving prednisone ≤ 30 mg/day and > 30 mg/day was compared. HCs healthy controls, SLE systemic lupus erythematosus, SLEDAI Systemic Lupus Erythematosus Disease Activity Index. *P < 0.05, **P < 0.01, ***P < 0.001, ****P < 0.0001; ns not significant

CircPTPN22 levels were significantly downregulated in 45 SLE patients compared to 44 HCs (Fig. 1A), and serum TNF-α and IL-6 levels were significantly higher in SLE patients than in HCs (Supplementary Fig. 1A and B). Patients with SLE with higher Systemic Lupus Erythematosus Disease Activity Index (SLEDAI) scores had lower circPTPN22 expression levels, both of which were negatively correlated (*r* =  − 0.781, *P* < 0.01) (Fig. 1B, C). To further assess the potential diagnostic value of circPTPN22, we compared its expression in patients with SLE with clinical and laboratory parameters, including skin involvement, alopecia, arthritis, lupus nephritis, leukopenia, anti-dsDNA ( +), anti-Sm ( +), C3 or C4 deficiency, and prednisone dose. The results showed that the expression of circPTPN22 in patients with SLE and alopecia, arthritis, lupus nephritis, anti-dsDNA ( +), and C3 or C4 deficiency was significantly lower than that in patients without these symptoms (all *P* < 0.05), but there was no significant difference between patients with skin involvement, leukopenia, anti-Sm ( +), and patients without these symptoms (Fig. 1D–K). In addition, circPTPN22 expression in the prednisone ≤ 30 mg/day group was significantly higher than that in the prednisone > 30 mg/day group (*P* < 0.05) (Fig. 1L). These results indicated that circPTPN22 may be a potential biomarker for SLE.

### CircPTPN22 enhances the proliferation and reduces the apoptosis of T cells

**Fig. 2:**
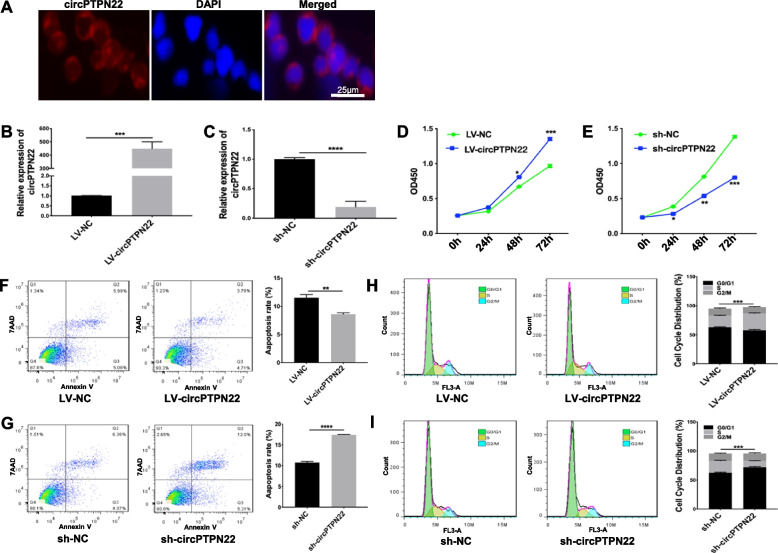
CircPTPN22 promoted Jurkat T-cell proliferation and cell cycle progression. **A** FISH assay to detect the subcellular localization of circPTPN22 in Jurkat T cells. The circPTPN22 probe was labelled with Cy-3. **B**, **C** Real-time PCR assay of circPTPN22 levels in Jurkat T cells transfected with LV-circPTPN22 or sh-circPTPN22. **D**,** E** CCK-8 assay to determine the proliferation of Jurkat T cells transfected with LV-circPTPN22 or sh-circPTPN22. **F**, **G** Flow cytometry analysis of apoptosis in Jurkat T cells transfected with LV-circPTPN22 or sh-circPTPN22. **H**, **I** Flow cytometry analysis of the proportion of cell cycle distribution in Jurkat T cells transfected with LV-circPTPN22 or sh-circPTPN22. The experiments were repeated three times. *P < 0.05, **P < 0.01, ***P < 0.001, ****P < 0.0001

To better understand the mechanism of circPTPN22 in T cells, we determined its subcellular localization in T cells using FISH. The results showed that circPTPN22 was mainly located in the cytoplasm of Jurkat T cells (Fig. 2A), suggesting that circPTPN22 may influence T-cell function by interacting with other proteins, polypeptides, or nucleic acids in the cytoplasm. The results further demonstrated that overexpression of circPTPN22 (Fig. 2B) led to significantly increased proliferation (Fig. 2D), decreased apoptosis (Fig. 2F), and accelerated cell cycle progression (Fig. 2H) in Jurkat T cells. In contrast, knockdown of circPTPN22 exerted the opposite effects on Jurkat T cells (Fig. 2C, E, G, and I).

### CircPTPN22 serves as a sponge for miR-4689

**Fig. 3:**
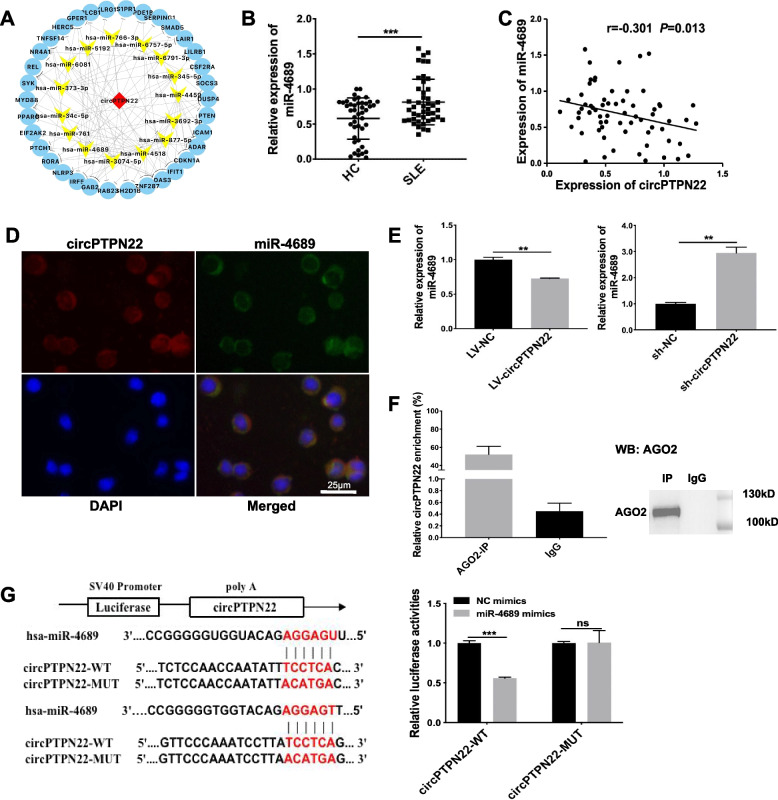
CircPTPN22 acts as a ceRNA for miR-4689. **A** CircPTPN22-miRNA‒mRNA interaction network constructed with Cytoscape software. **B** Relative expression of miR-4689 in PBMCs of 44 HCs and 45 patients with SLE. **C** Correlation analysis of circPTPN22 and miR-4689 expression levels. **D** Locations of circPTPN22 (red) and miR-4689 (green) were indicated by FISH in Jurkat T cells. The circPTPN22 probe was labelled with Cy-3, and the miR-4689 probe was labelled with FAM. Scale = 25 μm. **E** Real-time PCR assay for the expression of miR-4689 in Jurkat T cells transfected with LV-circPTPN22 or sh-circPTPN22. **F** RIP assay was performed in Jurkat T cells using anti-IgG or anti-AGO2 antibodies. The relative expression levels of circPTPN22 in immunoprecipitation were detected using real-time PCR, and the immunoprecipitated AGO2 protein was detected using western blot assay. **G** Prediction of the circPTPN22 binding sequence with miR-4689 and construction of the circPTPN22 MUT sequence. A luciferase reporter assay validated the binding relationship between circPTPN22 and miR-4689 in HEK-293 T cells. HCs healthy controls, SLE systemic lupus erythematosus, WT wild type, MUT mutant type. The experiments were repeated three times. **P < 0.01, ***P < 0.001; ns not significant

Because circRNAs can act as miRNA sponges, we constructed a circPTPN22-miRNA‒mRNA network based on our high-throughput RNA sequencing data, which showed that most miRNAs and mRNAs correlated with circPTPN22 are associated with immune regulation or have been reported as vital factors in the mechanism of SLE [13]. Interestingly, among these miRNAs, we found that miR-4689, a potential candidate miRNA target for circPTPN22 (Fig. 3A), was significantly increased in the PBMCs of SLE patients compared with that in HCs (Fig. 3B), which was negatively correlated with the expression of circPTPN22 (*r* =  − 0.301, *P* = 0.013) (Fig. 3C). We found that SLE patients with high SLEDAI scores had high miR-4689 expression levels, both of which were positively correlated (*r* = 0.808, *P* < 0.01), but there was no significant difference between the patients with a SLEDAI score of 0–4 and the patients with a SLEDAI score of 5–6 (Supplementary Figs. 2A and B). MiR-4689 has been reported to effectively inhibit Kirsten rat sarcoma virus (KRAS)-driven epidermal growth factor receptor (EGFR) signaling by directly inhibiting KRAS and AKT1 in colorectal cancer cells [14]. However, the mechanism by which miR-4689 functions in immune cells remains unknown. To further verify that circPTPN22 directly targets miR-4689 in immune cells, we performed FISH to determine circPTPN22 and miR-4689 localization in T cells and found that circPTPN22 and miR-4689 colocalized in the cytoplasm of Jurkat T cells (Fig. 3D). Furthermore, circPTPN22 overexpression decreased the expression of miR-4689, whereas circPTPN22 knockdown increased miR-4689 expression in Jurkat T cells (Fig. 3E).

RIP assays in Jurkat T cells showed that circPTPN22 was specifically enriched in immunoprecipitates pulled down by anti-AGO2 but not anti-IgG (Fig. 3F). The dual-luciferase reporter assay in HEK-293 T cells demonstrated that the relative luciferase activity was significantly reduced by cotransfection with circPTPN22 WT and miR-4689 mimics compared to that in the control group. Luciferase activity was not affected in the circPTPN22 MUT and miR-4689 mimic-cotransfected groups compared to that in the control group (Fig. 3G).

### S1PR1 is the target gene of miR-4689

**Fig. 4:**
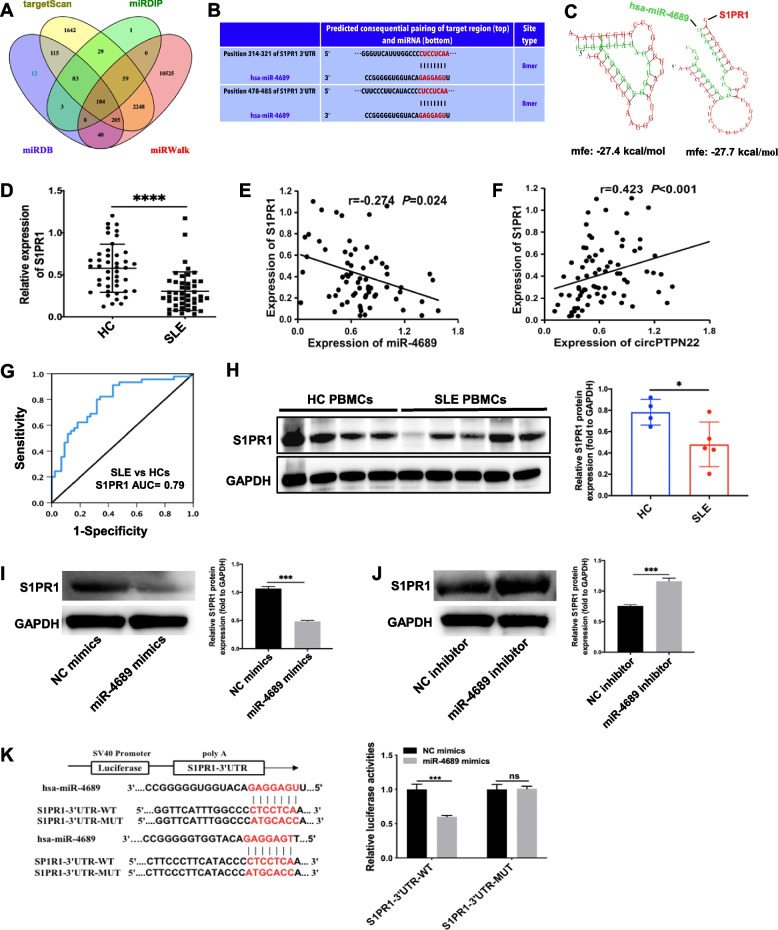
*S1PR1* is the target gene of miR-4689.** A** Venn diagram showing the common target genes of miR-4689 analysed using the TargetScan, miRDIP, miRDB, and miRWalk databases. **B** TargetScan indicated that miR-4689 had binding sites with S1PR1 in two positions. **C** Prediction of S1PR1 3'-UTR binding sites for miR-4689 using RNAhybrid tools. **D** The expression levels of S1PR1 mRNA in PBMCs from SLE patients and HCs were detected using real-time PCR. **E** Correlation analysis of miR-4689 and S1PR1 mRNA levels. **F** Correlation analysis of circPTPN22 and S1PR1 mRNA levels. **G**. ROC curve analysis of the sensitivity and specificity of S1PR1 in patients with SLE and HCs. **H** Western blotting results showing the protein expression level of S1PR1 in PBMCs from each group. **I**, **J** Expression levels of S1PR1 protein in Jurkat T cells transfected with miR-4689 mimics or inhibitor were determined by western blotting. **K** Binding sequence prediction of S1PR1-3' UTR-WT and miR-4689 and sequence construction of S1PR1-3' UTR-MUT. A luciferase reporter assay was used to validate the binding relationship between S1PR1 and miR-4689 in HEK-293 T cells. Mfe minimum free energy, SLE systemic lupus erythematosus, HCs healthy controls, ROC receiver operating characteristic, WT wild type, MUT mutant type. The experiments were repeated at least three times. *P < 0.05, ***P < 0.001, ****P < 0.0001; ns not significant

We used the TargetScan, miRDIP, miRDB, and miRWalk databases to predict the common target genes of miR-4689 and displayed them in a Venn diagram (Fig. 4A). Among the 104 candidate target genes, *S1PR1* was selected for further mechanistic exploration because of its pivotal role in immune regulation [15]. TargetScan indicated that miR-4689 has two perfect binding sites with the 3'-UTR of *S1PR1*, located at positions 314–321 and 478–485 (Fig. 4B). RNAhybrid predicted a strong combination ability of miR-4689 with *S1PR1* based on the minimum free energy (mfe) (Fig. 4C). In addition, remarkably downregulated *S1PR1* mRNA levels in the PBMCs of SLE patients were determined using qRT‒PCR (Fig. 4D). We found a negative correlation between miR-4689 and *S1PR1* mRNA expression (*r* =  − 0.274, *P* = 0.024) (Fig. 4E) and a positive correlation between circPTPN22 and *S1PR1* mRNA expression in PBMCs of patients with SLE (*r* = 0.423, *P* < 0.001) (Fig. 4F). In addition, receiver operating characteristic (ROC) curve analysis was used to validate the potential diagnostic value of *S1PR1* in SLE. As shown by the area under the curve (AUC) values, *S1PR1* expression could distinguish SLE patients from HCs, as expected (Fig. 4G). Meanwhile, the western blot assay showed that the S1PR1 protein expression level was markedly lower in SLE PBMCs than in HCs (Fig. 4H).

Functional experiments revealed that the expression of S1PR1 protein decreased in the miR-4689 mimic group and increased in the miR-4689 inhibition group compared with the NC groups in Jurkat T cells (Fig. 4I, J). Additionally, we constructed S1PR1-3' UTR-WT and S1PR1-3' UTR-MUT plasmids that were cotransfected into HEK-293 T cells with miR-4689 mimics or NC mimics. The results showed that luciferase activity was significantly decreased in cells cotransfected with S1PR1-3' UTR-WT and miR-4689 mimics compared with that in the control group. However, the luciferase activity in cells cotransfected with S1PR1-3' UTR-MUT and miR-4689 mimics was not significantly different from that in the control group (Fig. 4K).

### CircPTPN22 regulated S1PR1 expression by sponging miR-4689 to modify T-cell activation

**Fig. 5:**
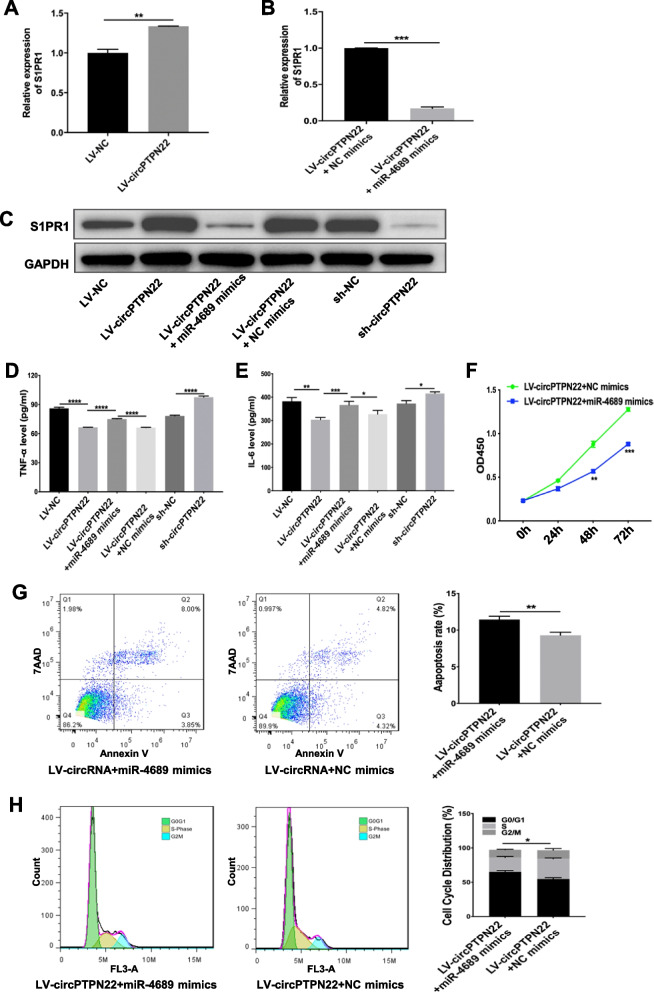
CircPTPN22-miR-4689 regulated T-cell activation via the S1PR1/STAT3 signaling pathway. **A**, **B** The expression of S1PR1 mRNA in Jurkat T cells from each group was detected using real-time PCR. **C** The protein expression of S1PR1 in Jurkat T cells from each group was detected using western blotting. **D**, **E** The levels of TNF-α and IL-6 in the supernatant of Jurkat T cells from each group were measured using an ELISA kit. **F** CCK-8 assay to determine the proliferation of Jurkat T cells transfected with LV-circPTPN22 and miR-4689 mimics or NC mimics. **G** Flow cytometry analysis of apoptosis in Jurkat T cells transfected with LV- circPTPN22 and miR-4689 mimics or NC mimics. **H** Flow cytometry analysis of the proportion of cell cycle distribution in Jurkat T cells transfected with LV-circPTPN22 and miR-4689 mimics or NC mimics. The experiments were repeated at least three times. *P < 0.05, **P < 0.01, ***P < 0.001, ****P < 0.0001

These results suggest that circPTPN22 functions in T cells by regulating S1PR1 expression by sponging miR-4689. We further found that the mRNA expression level of S1PR1 was increased in Jurkat T cells overexpressing circPTPN22 compared with that in the NC group (Fig. 5A), which could be rescued by miR-4689 mimics (Fig. 5B). CircPTPN22 overexpression promoted S1PR1 protein expression, whereas miR-4689 mimics significantly rescued this effect, and circPTPN22 knockdown markedly decreased S1PR1 protein expression in Jurkat T cells (Fig. 5C). Importantly, we demonstrated that circPTPN22 could modify the secretion of inflammatory cytokines by T cells. CircPTPN22 overexpression markedly decreased TNF-α and IL-6 secretion in Jurkat T cells that were stimulated with IL-2 protein, which could be rescued by miR-4689 mimics (Fig. 5D, E). Additionally, miR-4689 mimics partially reversed the effects of circPTPN22 on the proliferation, apoptosis, and cell cycle of Jurkat T cells (Fig. 5F–H). Furthermore, gene set enrichment analysis (GSEA) showed that the JAK-STAT signaling pathway was significantly enriched in differentially expressed mRNAs (Supplementary Fig. 3A). The PPI network constructed using the protein interaction prediction STRING software further demonstrated that S1PR1 could interact with STAT3 to activate downstream pathways (Supplementary Fig. 3B).

## Discussion

SLE has a poor prognosis owing to its unknown etiology. The data suggest that circRNAs play a crucial role in regulating autoimmunity or inflammation and participate in SLE development [16, 17]. In the present study, we confirmed that circPTPN22 is downregulated in the PBMCs of patients with SLE and explored the potential diagnostic value of circPTPN22 in SLE patients and its mechanisms of involvement in SLE. Finally, we found that circPTPN22 regulates S1PR1 by sponging miR-4689, which may be a potential therapeutic target for SLE.

Based on our previous studies, we found that circPTPN22 is downregulated in SLE PBMCs, and the association between the expression of circPTPN22 and the clinical and laboratory characteristics of SLE was verified. The SLEDAI is a global assessment of lupus disease activity generated by experienced clinicians that generates a “weighted” index of nine organ systems for disease activity in SLE [18]. SLEDAI scores determine the doses of hormones used and the selection of different immunosuppressive drugs [19]. In our results, SLE patients with a relatively high SLEDAI score received a higher dose of prednisone, while circPTPN22 expression was lower. Leukopenia, defined as a total white blood cell (WBC) count of < 4000/mm^3^ on two or more occasions, excluding drug causes, constitutes a deficiency of granulocytes and lymphocytes, but an absolute deficiency of granulocytes is usually found to be more severe than a deficiency of lymphocytes [20]. Zhang et al. showed no difference in the expression of hsa_circ0012919 in SLE patients with or without leukopenia [17]. This indicates that circPTPN22 has potential diagnostic value in SLE.

SLE is a complex, systemic autoimmune disease. T cells are chronically activated in SLE and can directly affect immune cells or secrete proinflammatory cytokines [21–23]. We verified that the expression levels of TNF-α and IL-6 are higher in SLE patients than in HCs. However, when we tried correlating the expression levels of TNF-α and IL-6 in SLE patients with circPTPN22, miR-4689, or S1PR1, we could not find any marked correlations (data not shown). This result may reflect the complexity of the factors that affect the production of cytokines in vivo. In particular, circPTPN22 functions in cytokine production should include several steps in vivo, i.e., circPTPN22 may first interact with target miRNAs, and the latter then inhibit the expression of the S1PR1 gene, which would further influence the expression of cytokines. Due to the long biological procedures for circPTPN22 effects on cytokine production and the fact that cytokines can be secreted by several kinds of immune cells and even tissue cells, we may not be able to observe the direct relationship between circPTPN22 and cytokine levels in vivo. Of course, the limited number of SLE patients in this study should contribute to the indeterminate correlation between circPTPN22 and cytokine levels in vivo.

Jurkat T cells are often selected as an in vitro biological model for T cells in SLE studies because their cellular phenotypes and functions are similar to those of normal T cells [24, 25]. CircRNAs are rich in miRNA-binding sites and can act as miRNA sponges to regulate the downstream expression of miRNA target genes by binding to miRNAs [26]. In this study, we explored the activation effect of circPTPN22 on Jurkat T cells and found that circPTPN22 promotes the proliferation and inhibits the apoptosis of Jurkat T cells. Furthermore, the production of TNF-α and IL-6 was reduced in Jurkat T cells overexpressing circPTPN22, which could be markedly rescued by miR-4689 transfection, suggesting that circPTPN22/miR-4689 plays a key role in modulating T-cell inflammation in SLE pathogenesis. Next, we found that miR-4689 was significantly increased in PBMCs from SLE patients compared to that in HCs. Bioinformatics analyses and dual-luciferase assays verified that circPTPN22 may serve as a sponge for miR-4689. To date, miR-4689 has only been reported in biliary atresia (BA) and mutant KRAS colorectal cancer [14, 27]. However, whether and how miR-4689 functions in T cells remains unknown. Our FISH assay showed that circPTPN22 colocalized with miR-4689 in Jurkat T cytoplasm. The dual-luciferase assay showed that circPTPN22 had a direct binding site for miR-4689, and the expression of miR-4689 was reduced when circPTPN22 was overexpressed. Collectively, these data suggest that circPTPN22 functions as a ceRNA for miR-4689 in T cells.

S1PR1 is involved in the development of various immune cells, mediates lymphocyte migration, and is abnormally expressed in various autoimmune diseases [28]. Under normal physiological conditions, there is a high concentration of S1PR1 in the peripheral blood and lymph [29, 30]. When inflammation occurs in the body, CD69, a C-type lectin, is expressed on the surface of immune cells and inhibits S1PR1 [31]. Xin et al. found that S1PR1 was downregulated in the PBMCs of patients with SLE and the spleen cells of lupus mice, and miR-155^(−/−)^ Fas^(lpr/lpr)^ mice inhibited the occurrence and development of SLE by regulating the target S1PR1 gene [32], indicating that S1PR1 is involved in the pathogenesis of SLE. In our study, S1PR1 was downregulated in the PBMCs of SLE patients, and the ROC curve indicated that S1PR1 might be a potential therapeutic target for SLE therapy. In Jurkat T cells, a dual-luciferase assay showed that S1PR1 directly binds to miR-4689 and that miR-4689 inhibits its expression. S1PR1 is involved in autoimmune diseases by directly or indirectly acting on STAT3 to activate downstream signaling pathways. In CD4 + T cells in Hashimoto thyroiditis, increased SphK1 produces more S1P, which activates the S1PR1-JAK2-STAT3 and S1PR1-mTOR-STAT3 pathways. Activated STAT3 amplifies the expression of inflammatory cytokines and S1PR1, which further exacerbates the development of Hashimoto thyroiditis [33]. Additionally, in rheumatic heart disease, overexpression of S1PR1 reduces p-STAT3 levels and Th17-associated cytokines RORγt, IL-6 and IL-17 [34]. Interestingly, in our study, GSEA based on our previous RNA sequencing data for SLE PBMCs [12] demonstrated that JAK-STAT signaling was enriched in differentially expressed mRNAs. PPI network prediction further demonstrated that S1PR1 could interact with STAT3 to activate downstream pathways. It is suggested that there are similar molecular mechanisms by which S1PR1 regulates downstream target genes in T cells in other immune diseases.

Although we showed the potential roles of the circPTPN22/miR-4689/S1PR1 axis in modulating T-cell functions using Jurkat T cells as a model, which are well-known CD4^+^ T cells, its functions in various immune cell subpopulations need to be further explored in the future to provide more exact mechanisms to fine-tune the immune response during SLE treatment. Moreover, animal models of SLE with genetic alterations could provide more potent evidence for the role of the circPTPN22/miR-4689/S1PR1 axis in SLE pathogenesis.

## Conclusions

We demonstrated that circPTPN22 regulates T-cell activation by sponging miR-4689 to target *S1PR1*, which might partially contribute to maladjusted T-cell functions in patients with SLE and thus provide a potential intervention target for SLE treatment.

### Supplementary Information

**Additional file 1: Supplementary Figure 1.** The expression of serum TNF-α and IL-6 in SLE patients and HCs. A and B. The levels of serum TNF-α and IL-6 in SLE patients and HCs were measured using ELISA. SLE, systemic lupus erythematosus; HCs, healthy controls; ***P<0.001.

**Additional file 2: Supplementary Figure 2.** The relationship between miR-4689 expression and SLEDAI score in SLE patients. A. SLEDAI score and miR-4689 expression in SLE patients. B. Correlation analysis of miR-4689 expression and SLEDAI score. SLEDAI, Systemic Lupus Erythematosus Disease Activity Index; SLE, systemic lupus erythematosus; ****P< 0.0001.

**Additional file 3: Supplementary Figure 3.** GSEA and PPI network predicted the interaction between S1PR1 and STAT3. A. GSEA was performed to identify JAK-STAT signaling pathways for differentially expressed mRNAs. B. STRING predicted the PPI network of S1PR1. GSEA, gene set enrichment analysis; NES, normalized enrichment score.

## Data Availability

The datasets used and/or analyzed in the study are available from the corresponding authors upon reasonable request.

## Abbreviations

CircRNAs: Circular RNAs
SLE: Systemic lupus erythematosus
S1PR1: 1-Sphingosine receptor 1
HCs: Healthy controls
CeRNAs: Competing endogenous RNAs
MiRNAs: MicroRNAs
3'-UTR: 3'-Untranslated region
RISC: RNA-induced silencing complex
PTPN22: Protein tyrosine phosphatase nonreceptor 22
PBMCs: Peripheral blood mononuclear cells
TNF-α: Tumor necrosis factor-alpha
IL-6: Interleukin-6
qRT‒PCR: Quantitative real-time PCR
CCK-8: Cell counting kit-8
7-AAD: 7-Aminoactinomycin D
PPI: Protein‒protein interaction
RIP: RNA-binding protein immunoprecipitation
AGO2: Argonaute-2
FISH: Fluorescent in situ hybridization
SLEDAI: Systemic lupus erythematosus disease activity index
Mfe: Minimum free energy
ROC: Receiver operating characteristic
AUC: Area under the curve
GSEA: Gene set enrichment analysis
