# Correction: CircPTPN22 modulates T‑cell activation by sponging miR‑4689 to regulate S1PR1 expression in patients with systemic lupus erythematosus

**DOI:** 10.1186/s13075-024-03270-4

**Published:** 2024-01-31

**Authors:** Zhuyan Jiang, Shifei Li, Yuying Jia, Qijun Wu, Xuemeng Chen, Mengjie Zhang, Qingqing Miao, Zhiting Zhong, Zhifang Zhai, Bing Ni, Jun Xiao, Jun Tang

**Affiliations:** 1 Dermatology Department of The First Affiliated Hospital of USTC, Division of Life Sciences and Medicine, University of Science and Technology of China, Hefei, Anhui 230001 China; 2 Department of Dermatology, Southwest Hospital, Third Military Medical University, Chongqing, 400038 China; 3 PLA Clinical College, Anhui Medical University, Hefei, Anhui 230001 China; 4 Department of Rheumatology, Southwest Hospital, Third Military Medical University, Chongqing, 400038 China; 5 Department of Pathophysiology, Third Military Medical University, Chongqing, 400038 China; 6 Intelligent Pathology Institute, Division of Life Sciences and Medicine, University of Science and Technology of China, Hefei, 230036 Anhui China; 7 Department of Cardiovascular Medicine, Chongqing University Central Hospital, Chongqing, 400014 China

**Correction: Arthritis Res Ther 25, 206 (2023)**

**https://doi.org/10.1186/s13075-023-03150-3**

Following publication of the original article [1], the authors reported an error found in the first affiliation. The “Division of Life Sciences and Medicine” should come after “The First Affiliated Hospital of USTC” and this is currently not correct. The affiliation should be: “Dermatology Department of The First Affiliated Hospital of USTC, Division of Life Sciences and Medicine, University of Science and Technology of China, Hefei, Anhui 230001, China”.

The original article [1] has been updated.
