# Supplementary figures and images for: CircPTPN22 modulates T-cell activation by sponging miR-4689 to regulate S1PR1 expression in patients with systemic lupus erythematosus
Source: Arthritis Res Ther. 2023 Oct 19;25:206. doi: 10.1186/s13075-023-03150-3 (PMC10585821; doi:10.1186/s13075-023-03150-3)

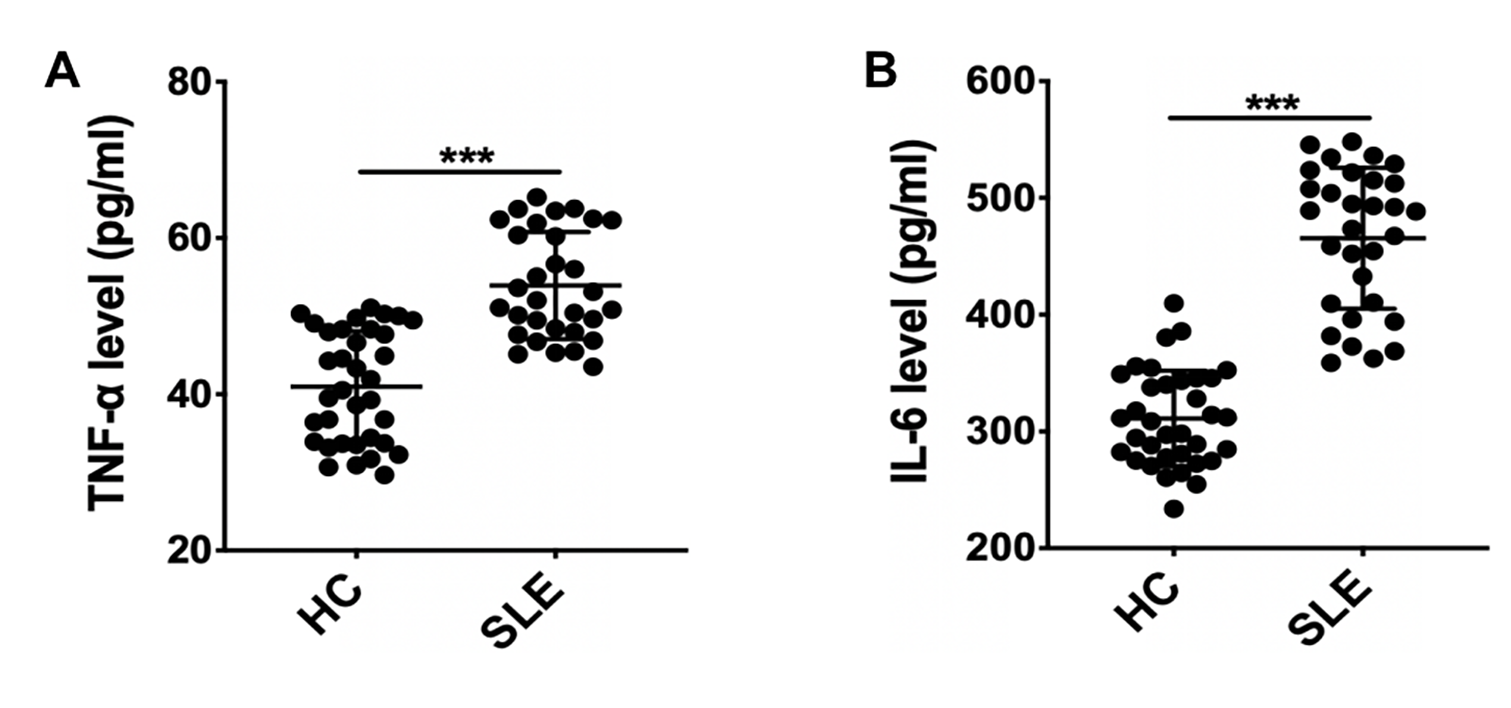

Supplement: Supplementary file 1 — Additional file 1: Supplementary Figure 1. The expression of serum TNF-α and IL-6 in SLE patients and HCs. A and B. The levels of serum TNF-α and IL-6 in SLE patients and HCs were measured using ELISA. SLE, systemic lupus erythematosus; HCs, healthy controls; ***P<0.001. [file 13075_2023_3150_MOESM1_ESM.tif]

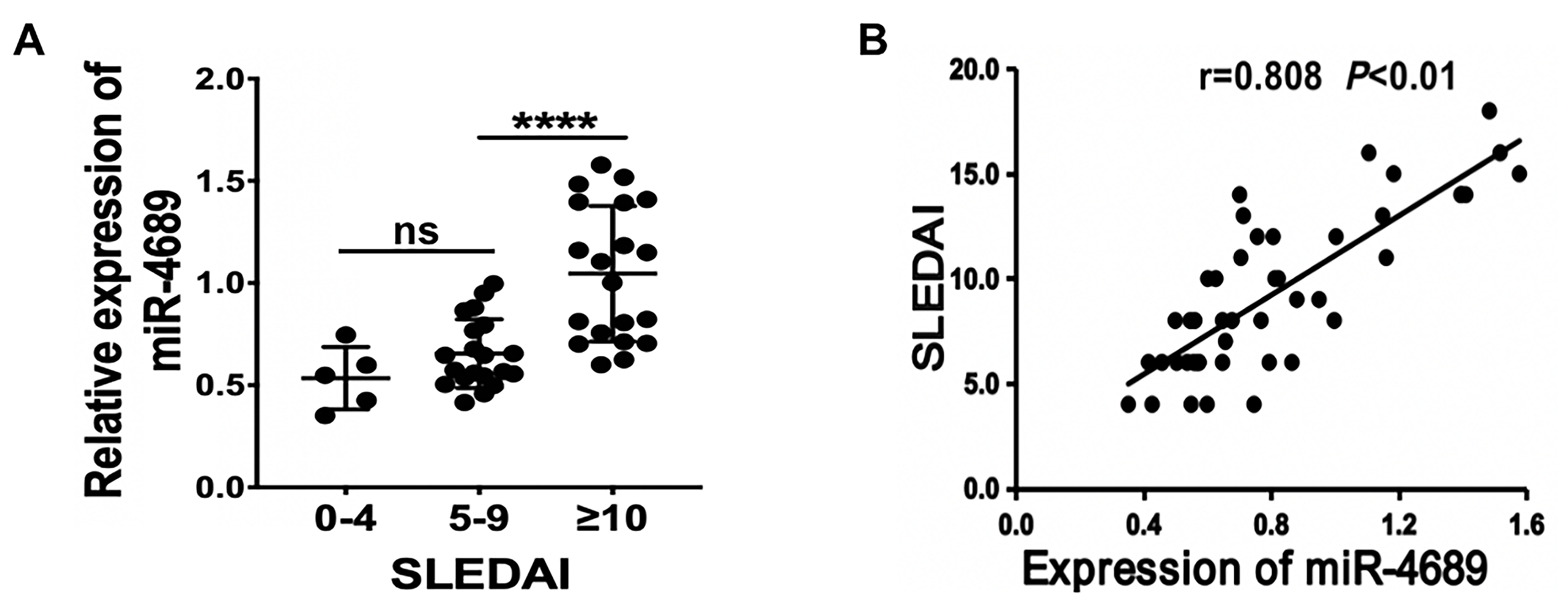

Supplement: Supplementary file 2 — Additional file 2: Supplementary Figure 2. The relationship between miR-4689 expression and SLEDAI score in SLE patients. A. SLEDAI score and miR-4689 expression in SLE patients. B. Correlation analysis of miR-4689 expression and SLEDAI score. SLEDAI, Systemic Lupus Erythematosus Disease Activity Index; SLE, systemic lupus erythematosus; ****P< 0.0001. [file 13075_2023_3150_MOESM2_ESM.tif]

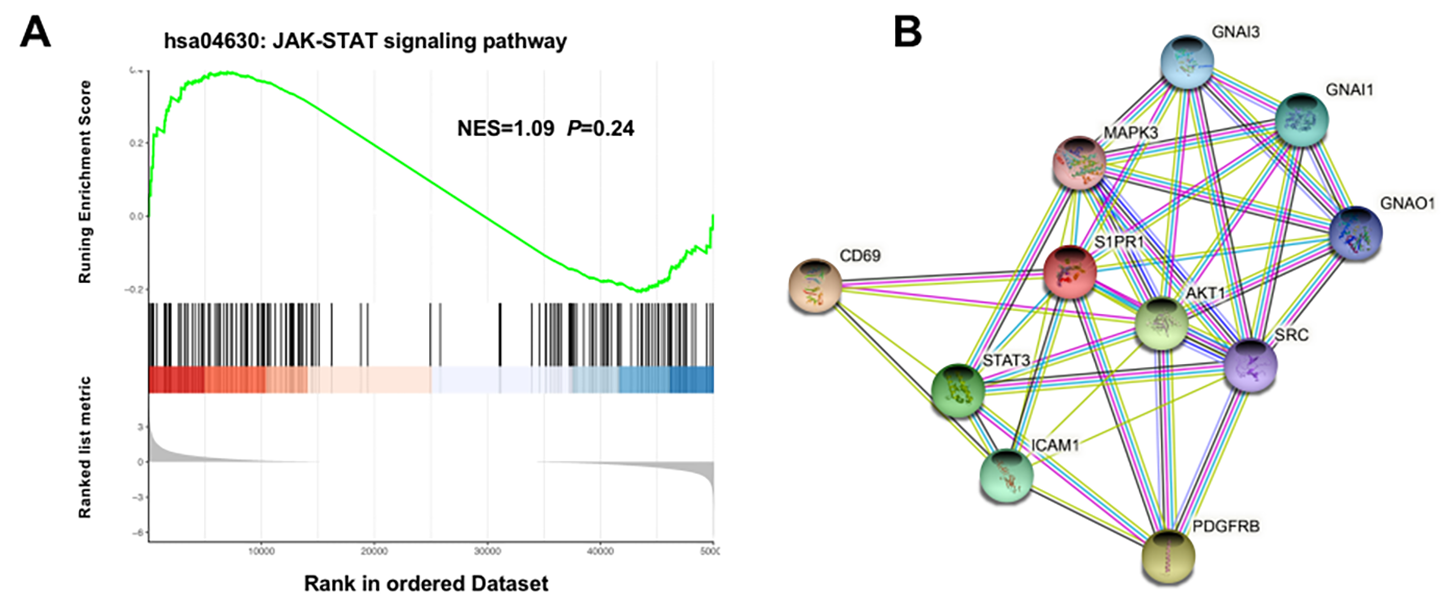

Supplement: Supplementary file 3 — Additional file 3: Supplementary Figure 3. GSEA and PPI network predicted the interaction between S1PR1 and STAT3. A. GSEA was performed to identify JAK-STAT signaling pathways for differentially expressed mRNAs. B. STRING predicted the PPI network of S1PR1. GSEA, gene set enrichment analysis; NES, normalized enrichment score. [file 13075_2023_3150_MOESM3_ESM.tif]
